# Transcriptome analysis of eutopic endometrial stromal cells in women with adenomyosis by RNA-sequencing

**DOI:** 10.1080/21655979.2022.2077614

**Published:** 2022-05-21

**Authors:** Lin Gan, Yongrong Li, Yan Chen, Meihua Huang, Jian Cao, Meiling Cao, Zhihui Wang, Guiping Wan, Tao Gui

**Affiliations:** a Department of Obstetrics and Gynecology, Affiliated Hospital of Integrated Traditional Chinese and Western Medicine, Nanjing University of Chinese Medicine, Nanjing, Jiangsu, China; b Department of Obstetrics and Gynecology, Suzhou Xiangcheng People’s Hospital, Suzhou, Jiangsu, China; c Laboratory of Obstetrics and Gynecology, Jiangsu Province Academy of Traditional Chinese Medicine, Nanjing, China; d Department of Gynecology, Women’s Hospital of Nanjing Medical University (Nanjing Maternity and Child Health Care Hospital), Nanjing, Jiangsu, China

**Keywords:** Adenomyosis, transcriptome, RNA sequencing, eutopic endometrial stromal cells, inflammatory cytokines, chemokines, angiogenesis

## Abstract

This study aimed to identify differentially expressed genes (DEGs) and molecular pathways in eutopic endometrial stromal cells (EuESCs) from adenomyosis (AM) patients and to provide a new insight into the disease mechanisms. The gene expression profiles in adenomyotic EuESCs (A-EuESCs) and normal ESCs (N-ESCs) were analyzed by RNA-sequencing (RNA-Seq) and validated by quantitative reverse transcription-polymerase chain reaction (qRT-PCR). Gene Ontology (GO) and Kyoto Encyclopedia of Genes and Genomes (KEGG) pathways enrichment analyses were performed to obtain insights into the functions of DEGs. The protein-protein interaction (PPI) network was constructed using the STRING database and visualized by Cytoscape software, and their hub genes were identified. A total of 458 up-/363 down-regulated genes were identified in A-EuESCs versus N-ESCs. The GO enrichment analysis showed that these genes were significantly enriched in calcium-dependent cell-cell adhesion. The most significant term of the KEGG pathway analysis was cytokine-cytokine receptor interaction. There were 145 nodes in the PPI network of the 157 DEGs, which were identified in significant enrichment pathway by the KEGG pathway analysis in N-ESCs and A-EuESCs. The PPI network revealed that IL-6 was a central hub gene. Besides, IL-6 was found as a central hub gene in the pro-inflammatory/chemotactic subnetwork, and EGF was noted as a central hub gene in the angiogenesis subnetwork. Our study indicated the alterations of transcriptomic profiles in A-EuESCs and provided new insights into the pathogenesis of AM. The A-EuESCs in women with AM have fundamental abnormalities that may predispose to pro-invasion/migration and angiogenesis.

## Graphical abstract

**Figure:**
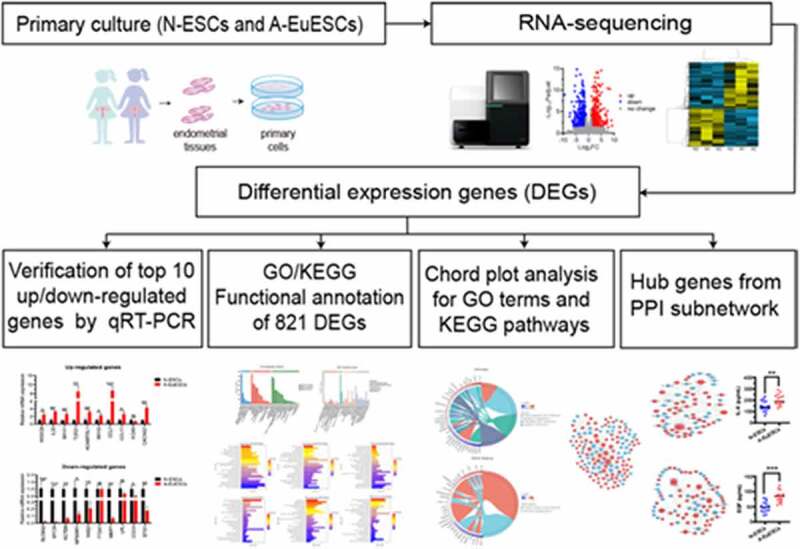

## Highlights

- 458 upregulated/363 downregulated genes were identified in A-EuESCs versus N-ESCs.
- IL-6 was found as a central hub gene in the pro-inflammatory/chemotactic subnetwork.
- EGF was noted as a central hub gene in the angiogenesis subnetwork.
- A-EuESCs may predispose to pro-invasion/migration and angiogenesis.

## Introduction

Adenomyosis (AM) is a common gynecological condition causing uterine enlargement, pelvic pain, menorrhagia and/or dysmenorrhea, in which and the condition is mainly refractory to drug treatments, and hysterectomy is sometimes essential for the complete alleviation of clinical symptoms [1,2]. Although several studies have concentrated on AM, but the etiology and pathogenesis of the AM have still remained elusive, which are worthy of further assessment [3,4].

As there is often a visualized histologic continuity between the ectopic and the basal endometrium in AM [5], it is widely accepted that AM results from the invagination of basalis endometrium into the myometrium through an altered or interrupted junctional zone, representing a highly specialized hormone-responsive structure located in the inner third of the myometrium [6–8]. Recent studies have suggested that a heritable or acquired alteration in the eutopic endometrium may play an essential role in the occurrence of AM [9–12]. Eutopic endometrium of the women with AM showed abnormal biological processes, including decreased apoptosis [8,13], increased proliferation [10] and angiogenesis [14], and impaired cytokine expression and local production of estrogens, which involved the pathogenesis of AM by enhancing the infiltration of the endometrium to the junctional zone and the growth of ectopic tissue [15]. Numerous AM-based studies have concentrated on the eutopic endometrium. Initially, gene expression profiles of endometrium from women with AM and age-matched healthy controls (HCs) had been explored with microarray platforms [16]. Subsequently, one study used RNA sequencing (RNA-Seq) to analyze differentially expressed genes (DEGs) that were in the eutopic endometrium of women with AM and HCs [17]. Recently, single-cell RNA sequencing (scRNA-seq) has been applied to identify the changes in gene expression patterns among ectopic lesions, eutopic endometrium, and normal endometrium at the single-cell level and to explore a potential novel pathogenesis of AM [18]. However, these studies were carried out without separating the endometrial stromal cells (ESCs) from glandular epithelial cells in eutopic endometrial tissue.

Invasion of abnormal adenomyotic eutopic endometrial stromal cells (A-EuESCs) has been reported in the etiology of AM [19,20]. The stromal cells may play a primary pathogenetic role in accelerating epithelial downgrowth [6]. In addition, both exogenous and endogenous interleukin-22 (IL-22) have enhanced the invasiveness of A-EuESCs *in vitro* [21]. Besides, A-EuESCs were proliferated more rapidly than normal endometrial stromal cells (N-ESCs), whether they were treated with or without estradiol (E2), medroxyprogesterone acetate (MPA), interleukin 6 (IL-6), lipopolysaccharide (LPS) and interferon γ (IFN-γ) [10,14]. A-EuECSs also expressed a higher level of AMP-activated protein kinase (AMPK) than N-ESCs [22]. As described above, studies on A-EuESCs were limited to the evaluation of expression levels of one or several particular genes [13,23,24], while few systematic studies have concentrated on the DEGs or the major pathways involved in AM from the perspective of A-EuESCs [25]. Hence, identifying distinct gene expression profiles in A-EuESCs and N-ESCs is of great significance to better understand the pathogenesis of AM.

As mentioned earlier, A-EuESCs exhibited dysregulation of pathways that globally predispose toward the development, invasion/migration, and survival of ectopic endometrial implants beyond the myometrial interface. It is reasonable to postulate the existence of intrinsic abnormalities in A-EuESCs from women with AM. In the present study, we performed the transcriptome analysis of A-EuESCs from women with or without AM by RNA-Seq, and aimed to identify DEGs and molecular pathways/networks in A-EuESCs and to provide new insights into underlying mechanisms of AM.

## Materials and methods

### Patients and tissue sample collection

Female patients with AM and women without AM as age-matched HCs were enrolled in the present study. All patients had not received hormone therapy or used intrauterine contraceptive device for at least 6 months prior to surgery. Eutopic endometrial tissues were collected by hysterectomy from symptomatic women with pathologically confirmed diffuse AM, and also from female age-matched HCs by hysterectomy who had no endometrial disorders (e.g., intramural myomas) and pathologically confirmed to be free of AM or endometriosis.

All eutopic endometrial tissues were collected on the days of 20–23 of the menstrual cycle (middle-secretory phase) for isolation and culture of primary ESCs [26]. The study was approved by the Ethic Committee of Integrated Traditional Chinese and Western Medicine Hospital of Jiangsu Province (Nanjing, China; Approval No. 2019LWKYS-001). Informed consent was obtained from all participants prior to enrollment.

### Primary cell culture

The isolation and culture of A-EuESCs and N-ESCs were carried out based on previously described procedures with slight modification [27]. Briefly, endometrial samples obtained by surgery were immediately placed in ice-cold sterile phosphate-buffered saline (PBS) and transferred to the laboratory. Tissues were thrice washed with sterile PBS and minced into small pieces, and then incubated with 0.1% (w/v) collagenase type II (Sigma-Aldrich, St. Louis, MO, USA) in a shaking water bath for 0.5 h at 37°C. The cell suspension was sequentially filtered through a 100-μm filter, and then, through a 40-μm cell strainer (BD Falcon, Bedford, MA, USA), followed by removing the debris and epithelial cells, respectively. The cell suspension was collected and centrifuged at 200 × g for 5 min to obtain ESCs. The pellet was re-suspended in a Dulbecco’s modified Eagle’s medium (DMEM)/F12 (1:1) (Invitrogen, Carlsbad, CA, USA) containing 10% fetal bovine serum (FBS) (Gibico, New York, NY, USA) and 1% penicillin/streptomycin (Invitrogen), 10 nmol/L 17-estradiol (Sigma-Aldrich), and 1 umol/L medroxyprogesterone acetate (MPA) (Sigma-Aldrich) [28]. Cells were seeded at a density of 2 × 10^5^ cells per T25 flask and incubated in a 5% CO_2_ atmosphere at 37°C. The cultured ESCs were identified by immunocytochemical staining for vimentin and cytokeratin 8 as previously described (data were not shown) [29].

### RNA-seq and data analysis

To preserve the biological properties of the ESCs, RNA-Seq was performed using primary cells (all passage 1) when the cell confluence reached 80% at 6–7 days after culture. Total RNA was extracted from cultured cells using TRIzol® reagent (Invitrogen) according to the manufacturer’s instructions. RNA-Seq transcriptome library was prepared through a TruSeqTM RNA sample preparation kit (Illumina Inc., San Diego, CA, USA) using 1 μg of total RNA [30,31]. Then, mRNA sequencing was performed on an Illumina NovaSeq 6000 platform (Illumina Inc.) by Shanghai Majorbio Bio-pharm Technology Co.,Ltd (Shanghai, China). To identify DEGs in two different groups, the expression level of each transcript was calculated according to transcripts per million (TPM) reads. RSEM software was used to quantify gene abundances. DESeq2 R package (ver. 1.22.2) was utilized for annotation and differential expression analysis. DEGs were identified with adjusted *P* < 0.05 and absolute Log_2_ fold-change (FC) ≥ 1. Gene Ontology (GO) and Kyoto Encyclopedia of Genes and Genomes (KEGG) pathways enrichment analyses were performed to identify significant biological processes and pathways [32,33], using Goatools and KOBAS, respectively, with *P*-value < 0.05.

In order to explore the relationship between key genes and GO terms/KEGG pathways at a clearer glance, chord plots of GO terms and KEGG pathways were also drawn. The data were analyzed using Majorbio cloud platform (www.majorbio.com). The data discussed in the present study were deposited in the Gene Expression Omnibus (GEO) database (Accession No. GSE157718) [34].

### Quantitative reverse transcription polymerase chain reaction (qRT-PCR)

Total RNA was extracted from the ESCs (all passage 2) using TRIzol® reagent as described previously. The cDNA was synthesized using the a HiScript®III Reverse Transcriptase kit (Vazyme Biotech Co., Ltd., Nanjing, China) for qRT-PCR. The thermocycling conditions of the reverse transcription were as follows: Removing genomic DNA by gDNA wiper at 42 °C for 2 min; synthesizing first- strand cDNA at 25 °C for 5 min, 37 °C for 45 sec, and at 85 °C for 5 sec. qPCR was subsequently performed using an AceQ qPCR SYBR Green Master Mix system (Vazyme Biotech Co., Ltd.) according to the manufacturer’s instructions. Experiments were performed by a QuantStudio™ 5 Real-Time PCR system (Applied Biosystems, Waltham, MA, USA). The thermocycling conditions of the qPCR were as follows: pre-denaturation at 95 °C for 5 min; 40 cycles at 95 °C for 10 sec, and at 60 °C for 30 sec; and the final dissociation stage (at 95 °C for 15 sec, at 60 °C for 60 sec and at 95 °C for 15 sec) was performed at the end of the amplification procedure. The relative mRNA expression levels were normalized to glyceraldehyde 3-phosphate dehydrogenase (GAPDH), as a

reference gene, and calculated using the 2^‑ΔΔCT^ method [35,36]. The qRT-PCR reactions were performed in triplicate in 96-well optical reaction plates. The GraphPad Prism 9.0 software package (GraphPad Software Inc., San Diego, CA, USA) was used to draw figures. The primers used for RT-qPCR are listed in Supplementary Table 1.

### Construction of protein-protein interaction (PPI) network

The Search Tool for the Retrieval of Interacting Genes (STRING) database (ver. 11.0; https://string-db.org/) was used to elucidate the interactive relationships of the DEGs identified in significantly enriched pathways (adjusted *P* < 0.05) by the KEGG pathway analysis [37]. The interacting pairs with a confidence score greater than 0.4 were considered significant and were retained. Subsequently, Cytoscape software (ver. 3.8.1) was used to establish the PPI network [38]. The network topology property indicators, including degree centrality, betweenness centrality, and closeness centrality were analyzed using CytoNCA in Cytoscape software. A node with a higher score of network topology property indicators indicated a more important role in that node in the PPI network, which was considered as a hub node [39].

### Cytokine assay

N-ESCs (n = 15) and A-EuESCs (n = 15) at passage 2 were cultured at a density of 5 × 10^5^ cells per a 60-mm dish. The conditioned medium from each dish was collected after 48 h of inoculation, then, centrifuged at 2,500 rpm for 5 min at room temperature, and the culture supernatants were filtered through a 0.22-μm pore-sized filter and stored at −80°C. The levels of interleukin-6 (IL-6) and epidermal growth factor (EGF) in the supernatants were determined using enzyme-linked immunosorbent assay (ELISA) kits (Elabscience Biotechnology Co., Ltd., Wuhan, China). The measurements were performed according to the manufacturer’s instructions [40]. Each experiment was carried out in triplicate and repeated three times.

### Statistical analysis

The statistical analysis was performed using SPSS 22.0 software (IBM Corp., Armonk, NY, USA). Continuous variables were presented as mean ± standard deviation (SD). Categorical data were expressed as number (percentage). The Chi-square and test or the Fisher’s exact test, whatever appropriate, were used for data analysis. The independent two-sample t-test or the Mann-Whitney-U test, whatever appropriate, were utilized to compare the continuous variables between the two groups. *P* < 0.05 was considered statistically significant.

## Results

### Clinical characteristics in the AM and control groups

A total of 18 female patients with AM and 18 age-matched female HCs were enrolled in our study. All patients’ endometrial tissues were collected by hysterectomy during the secretory phase. Besides, 18 patients with AM were multipara who aged 32 to 53 (median age, 41.6) years old; 18 age-matched female HCs were multipara who aged 34 to 55 (median age, 42.3) years old. None of the age-matched female HCs suffered from AM. The clinical data of participants are presented in Table 1.

**Table 1.** Patients characteristics and symptoms of the study population

Variable	Control (n = 18)	AM (n = 18)	*P*-value
Age, years			
mean ± SD	41.6 ± 3.78	42.3 ± 4.16	0.271
Body mass index (BMI), kg/m^2^			
mean ± SD	20.9 ± 2.63	21.4 ± 3.19	0.347
Gravidity, n (%)			
0	1 (5.6%)	2 (11.1%)	0.546
≥1	17 (94.4%)	16 (88.9%)	
Parity, n (%)			
0	2 (11.1%)	3 (16.7%)	0.630
≥1	16 (88.9%)	15 (83.3%)	
Menorrhagia, n (%)			
Yes	0 (0.0%)	12 (66.7%)	0.000
No	18 (100.0%)	6 (33.3%)	
Dysmenorrhea, n (%)			
Yes	0 (0.0%)	15 (83.3%)	0.000
No	18 (100.0%)	3 (16.7%)	
Previous miscarriages, n (%)			
0	14 (77.8%)	13 (72.2%)	0.700
≥1	4 (22.2%)	5 (27.8%)	

### RNA-Seq analysis and identification of DEGs

**Figure 1.**
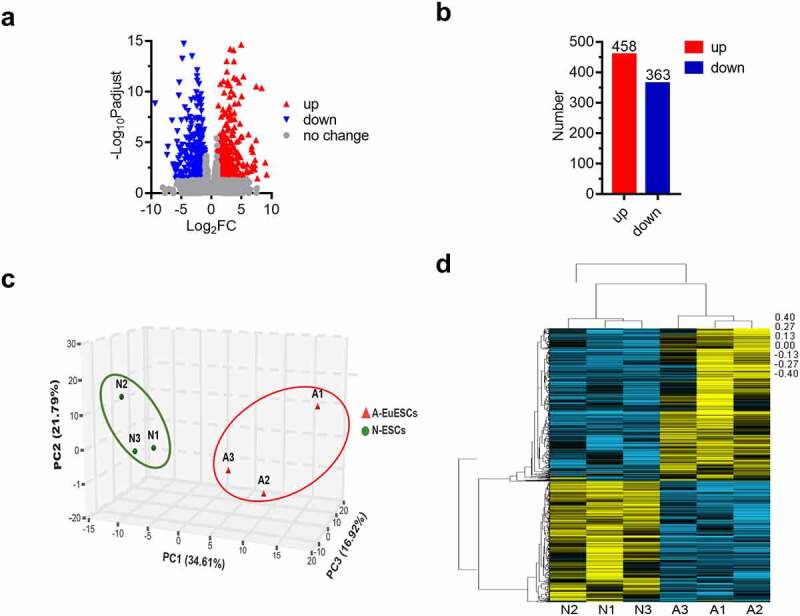
Differential expression analysis in N-ESCs and A-EuESCs (a) Volcano plot shows DEGs in N-ESCs and A-EuESCs. (b) Bar chart showed the number of upregulated and downregulated DEGs in A-EuESCs compared with N-ESCs. (c) Unsupervised principal component analysis (PCA) of different cell lines (3 N-ESCs and 3 A-EuESCs). (d) Hierarchical clustering analysis of the DEGs in N-ESCs group (3 N-ESCs are abbreviated as N1, N2, and N3, respectively) and A-EuESCs group (3 A-EuESCs are abbreviated as A1, A2, and A3, respectively). Yellow and blue bar charts represent significantly upregulated and downregulated genes, respectively.

To better understand the molecular mechanism of AM, we conducted a comparative transcriptomic analysis on ESCs from 3 AM and 3 age-matched female HCs. Totally, 29655 annotated mRNAs were identified, of which 821 mRNAs (458 upregulated and 363 downregulated) were significantly deregulated (Figure 1(a,b)). The top 10 genes that were significantly upregulated or downregulated are listed in Table 2. The principle component analysis (PCA) showed that the A-EuESCs exhibited distinct gene expression profiles compared with N-ESCs (Figure 1(c)). Hierarchical cluster analysis of the DEGs in ESCs from AM and control groups indicated that the gene expression patterns were clustered separately after unsupervised clustering (Figure 1(d)). The above-mentioned results suggested that the gene expression profiles in A-EuESCs in AM group were significantly altered compared with N-ESCs in control group.

**Table 2.** The DEGs in the A-EuESCs compared to N-ESCs

Gene Symbol	Gene ID	*P*-value	*P*-adjust	log_2_FC
Up-regulation genes (Top 10)				
HOXC8	3224	1.53E-39	0.00094	8.97
IL33	90865	2.59E-32	4.27E-11	8.37
MYH1	4619	1.44E-31	0.036	7.67
TCF21	6943	6.44E-24	2.98E-11	7.48
ADAMTSL1	92949	8.43E-24	5.95E-06	7.43
MYH2	4620	1.4E-23	0.00054	7.29
CCL7	6354	1.36E-22	0.0017	7.23
CCL11	6356	2.27E-22	0.0045	7.21
ACAN	176	3.61E-22	0.0037	6.92
CACNG7	59284	1.49E-20	8.66E-05	6.90
Down-regulation genes (Top 10)				
SLC6A2	6530	4.59E-12	1.5E-09	−9.34
MYCN	4613	2.75E-07	2.8E-05	−7.22
KCTD8	386617	3.39E-10	7.34E-08	−6.53
NPBWR1	2831	5.81E-05	0.0025	−6.26
NKD2	85409	0.000112	0.0042	−6.07
F13A1	2162	0.000267	0.0083	−5.91
MMP7	4316	0.002097	0.041	−5.90
LPL	4023	0.001222	0.026	−5.80
COCH	1690	5.33E-66	9.95E-62	−5.75
DTX1	1840	0.0015	0.030	−5.71

FC: fold change

### Verification of the mRNA expression levels of DEGs

**Figure 2.**
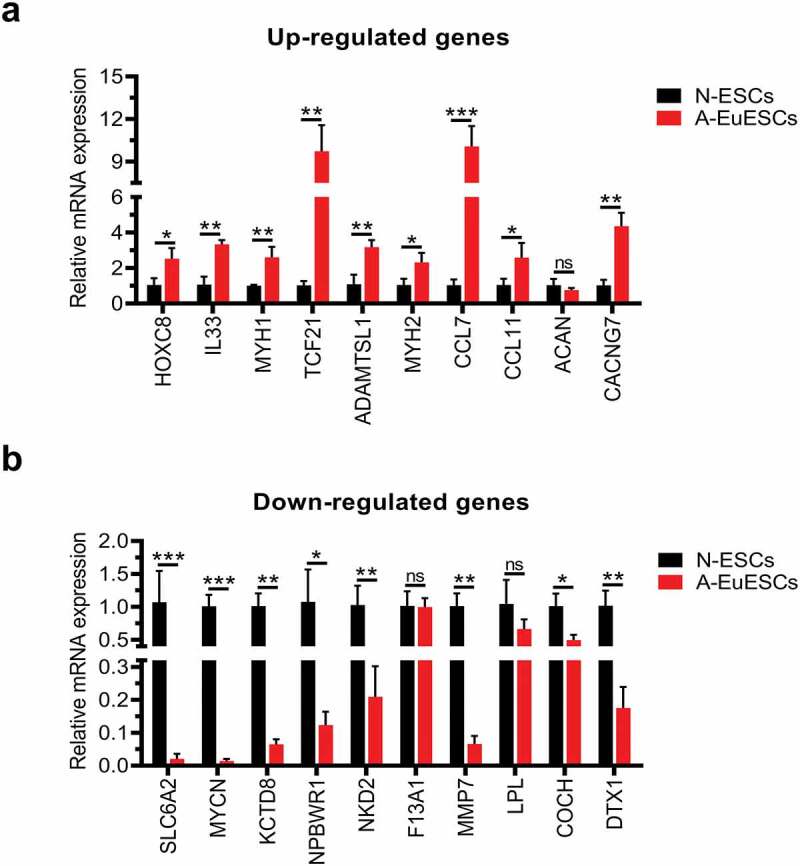
Verification of the expression levels of DEGs by qRT-PCR qRT-PCR of top 10 upregulated (a) or downregulated (b) DEGs by RNA-Seq in A-EuESCs (n = 15) compared with N-ESCs (n = 15). GAPDH was used as an internal control for normalization. At least three independent experiments were performed for statistical evaluation. Error bars represent standard deviation (SD). *P < 0.05, *P < 0.01, *P < 0.001, A-EuESCs *vs*. N-ESCs; ns, not statistically significant.

To confirm the accuracy of RNA-Seq, the top 10 upregulated and top 10 downregulated genes in N-ESCs (n = 15) and A-EuESCs (n = 15) were selected for further validation by qRT-PCR in an additional set of endometrial tissues. It was revealed that *HOXC8* (*P* < 0.05), *IL-33* (*P* < 0.01), *MYH1* (*P* < 0.01), *TCF21* (*P* < 0.01), *ADAMTSL1* (*P* < 0.01), *MYH2* (*P* < 0.01), *CCL7* (*P* < 0.001), *CCL11* (*P* < 0.05) and *CACNG7* (*P* < 0.01) genes were significantly up-regulated in A-EuESCs compared with N-ESCs (Figure 2(a)), and the expression level of *ACAN* (*P* = 0.25) did not significantly differ between N-ESCs and A-EuESCs. Moreover, *SLC6A2* (*P* < 0.001), *MYCN* (*P* < 0.001), *KCTD8* (*P* < 0.01), *NPBWR1* (*P* < 0.05), *NKD2* (*P* < 0.01), *COCH* (*P* < 0.05), and *DTX1* (*P* < 0.01) genes were significantly down-regulated in A-EuESCs compared with N-ESCs (Figure 2(b)), and the expression levels of *F13A1* (*P* = 0.90) and *LPL* (*P* = 0.17) did not significantly differ between N-ESCs and A-EuESCs. Meanwhile, the other upregulated genes including *ADAM12, SERPINE1, ADGRA2, ANGPTL4, IL6, NTF3, CCL2, TLR3, GATA6, HGF*, and *KDR* were also selected for validation, which are shown in Supplementary Figure 1, and the validation results achieved by qRT-PCR were almost identical to the RNA-Seq results.

### Functional annotation analysis of DEGs

**Figure 3.**
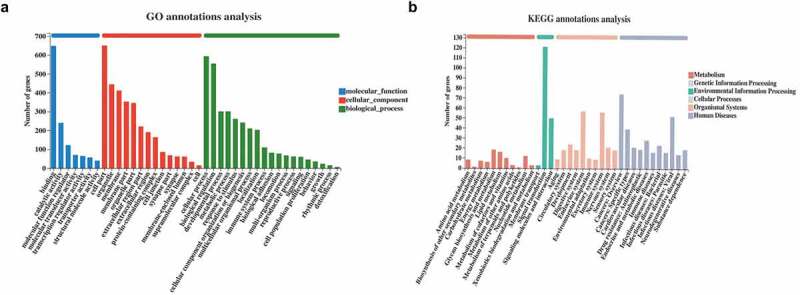
Functional annotation analysis of DEGs (a) GO annotation was performed using 821 DEGs in N-ESCs and A-EuESCs to obtain insights into their biological functions. The DEGs were classified into three functional groups, including biological process, cellular component and molecular function. (b) KEGG annotation was performed using 821 DEGs in N-ESCs and A-EuESCs to obtain insights into their pathways involved.

GO annotation was performed on 821 DEGs in N-ESCs and A-EuESCs to obtain insights into their biological functions. Binding (GO:0005488), catalytic activity (GO:0003824), and molecular function regulator (GO:0098772) were the most enriched molecular functions. Cell part (GO:0044464), organelle (GO:0043226), and membrane (GO:0016020) were the most enriched cellular components. Cellular process (GO:0009987), biological regulation (GO:0065007), and developmental process (GO:0032502) were the most enriched biological processes (Figure 3(a)). KEGG annotation was performed on 821 DEGs in N-ESCs and A-EuESCs to obtain insights into their pathways involved. The first three pathways with the highest number of DEGs were signal transduction (n = 121), cancers: overview (n = 74) and endocrine system (n = 57) (Figure 3(b)).

### Functional enrichment analysis of DEGs

**Figure 4.**
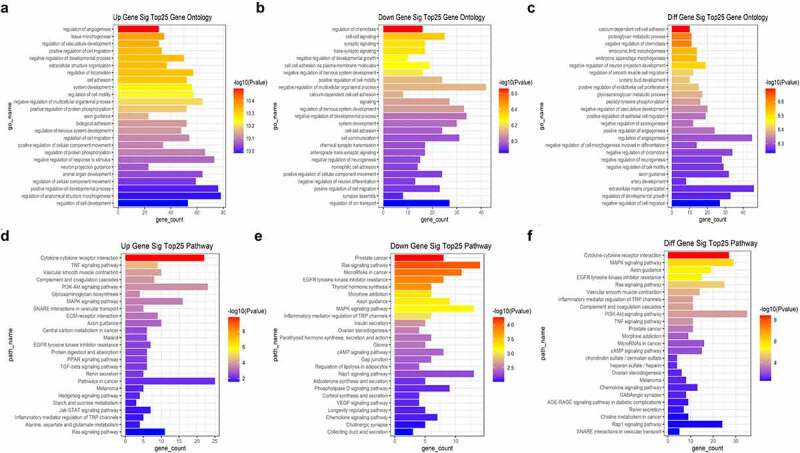
GO and KEGG pathway enrichment analyses of the significant DEGs. (a) The top 25 enriched GO biological processes of the upregulated DEGs. (b) The top 25 enriched GO biological processes of the downregulated DEGs. (c) The top 25 enriched GO biological processes of all DEGs. (d) The top 25 enriched KEGG pathways of the upregulated DEGs. (e) The top 25 enriched KEGG pathways of the downregulated DEGs. (f) The top 25 enriched KEGG pathways of all DEGs.

To define the biological functions of the 821 DEGs, GO and KEGG pathway enrichment analyses were performed. The GO term enrichment analysis showed that the up-regulated DEGs were significantly enriched in regulation of angiogenesis (GO:0045765), tissue morphogenesis (GO:0048729) and regulation of vasculature development (GO:1901342) (Figure 4(a)). Meanwhile, the down-regulated DEGs were significantly enriched in regulation of chemotaxis (GO:0050920), cell-cell signaling (GO:0007267), and synaptic signaling (GO:0099536) (Figure 4(b)). The functions of all DEGs were significantly enriched in calcium-dependent cell-cell adhesion (GO:0016339), proteoglycan metabolic process (GO:0006029), and negative regulation of chemotaxis (GO:0050922) (Figure 4(c)).

The KEGG pathway analysis revealed that the up-regulated DEGs were significantly enriched in cytokine-cytokine receptor interaction (map04060), TNF signaling pathway (map04668) and vascular smooth muscle contraction (map04270) (Figure 4(d)), while the down-regulated DEGs were significantly enriched in prostate cancer (map05215), Ras signaling pathway (map04014) and microRNAs in cancer (map05206) (Figure 4(e)). The KEGG enrichment analysis showed that all the DEGs were mostly significantly enriched in cytokine-cytokine receptor interaction (map04060), MAPK signaling pathway (map04010), and axon guidance (map04360) (Figure 4(f)).

### Analysis of chord plot

**Figure 5.**
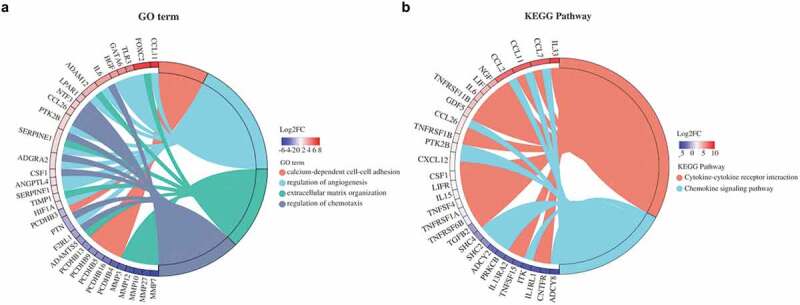
Chord plot analysis of GO terms and KEGG pathways. (a) GO chord plot of 4 designated GO terms belonging to the Biological Process (BP) sub-ontology for A-EuESCs. (b) KEGG chord plot of 2 designated KEGG pathways for A-EuESCs. The genes are linked to their assigned terms or pathways via colored ribbons. Genes are ordered according to the observed log2 fold-change (log2FC), which can be shown in descending intensity of red squares next to the selected genes.

The four designated GO terms, including calcium-dependent cell-cell adhesion, regulation of angiogenesis, extracellular matrix organization, and regulation of chemotaxis, belong to biological process (BP) subontology. The presence of IL-6, LPAR1, NTF3, CCL26, and PTK2B was directly related to the regulation of chemotaxis (Figure 5(a)). Importantly, the upregulated transcripts of forkhead box protein C2 (FOXC2) and hypoxia-inducible factor 1A (HIF1A) were involved in the regulation of angiogenesis (Figure 5(a)). Several MMP family genes (*MMP3, MMP7, MMP10, MMP12* and *MMP27*), with a particularly pronounced down-regulation in A-EuESCs, were found to be associated with extracellular matrix organization (Figure 5(a)). Furthermore, we observed a significant downregulation of protocadherin-beta (PCDHB) family genes (*PCDHB3, PCDHB4, PCDHB5, PCDHB9, PCDHB13*, and *PCDHB16*) in A-EuESCs, which were associated with calcium-dependent cell-cell adhesion (Figure 5(a)). The KEGG pathway analysis revealed that a significantly upregulated chemokine family member genes (*CCL2, CCL7, CCL11, CCL26*, and *CXCL12*) were associated with chemokine signaling pathway (map04062) (Figure 5(b)). In addition to the above-mentioned chemokines, several pronounced upregulated proinflammatory cytokines (*IL-33, LIF, IL-6* and *IL-15*) in A-EuESCs were involved in cytokine-cytokine receptor interaction (map04060) (Figure 5(b)).

### PPI networks of the DEGs in N-ESCs and A-EuESCs

**Figure 6.**
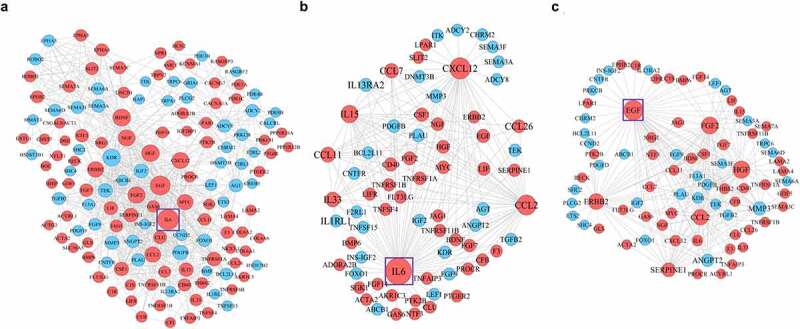
The constructed PPI networks and subnetworks of the DEGs in N-ESCs and A-EuESCs. (a) PPI network of the 157 DEGs identified in significant enrichment pathway (adjusted *P* < 0.05) by the KEGG pathway analysis in N-ESCs and A-EuESCs. (b) PPI subnetwork of the DEGs related to inflammatory cytokines and chemokines. (c) PPI subnetwork of the DEGs related to the regulation of angiogenesis. The red circular nodes represent the upregulated DEGs and the blue circular nodes represent the downregulated DEGs; black edges indicate the interaction between two proteins. The larger the circular nodes, the greater the centrality in the PPI network.

PPI networks of the 157 DEGs identified in significant enrichment pathway (adjusted *P* < 0.05) by the KEGG pathway analysis in N-ESCs and A-EuESCs were constructed using the STRING database and visualized by the Cytoscape software. In the PPI networks of 157 DEGs, there were 145 nodes and 793 edges (Figure 6(a)). According to the ranking of network topology property indicator of degree centrality, the top 10 nodes were separately identified as hub genes, and IL-6 was a central hub gene in the network, with the maximum number of degree (n = 61) (see Table 3). Besides, PPI subnetwork of the DEGs related to inflammatory cytokines and chemokines were constructed as described above. In these PPI subnetworks, there were 71 nodes and 177 edges (Figure 6(b)). As mentioned earlier, the top 10 nodes were separately identified as hub genes (Table 4), and IL-6 was also a central hub gene in the subnetworks (n = 61) (Table 4). Moreover, another subnetwork of the DEGs related to the regulation of angiogenesis was constructed, including 82 nodes and 240 edges (Figure 6(c)). Similarly, the top 10 nodes were also identified as hub genes (Table 5), and EGF was a central hub gene in this subnetwork (n = 55).

**Table 3.** The hub nodes in the PPI network according to the score of network topology property indicators

Rank	Node	Locus	Gene description	Degree
1	IL6	7p15.3	interleukin 6	61
2	EGF	4q25	epidermal growth factor	55
3	BDNF	11p14.1	brain derived neurotrophic factor	43
4	HGF	7q21.11	hepatocyte growth factor	42
5	KDR	4q12	kinase insert domain receptor	41
6	FGF2	4q28.1	fibroblast growth factor 2	40
7	CXCL12	10q11.21	C-X-C motif chemokine ligand 12	38
8	NGF	1p13.2	nerve growth factor	38
9	ERBB2	17q12	erb-b2 receptor tyrosine kinase 2	34
10	CCL2	17q12	C-C motif chemokine ligand 2	33

**Table 4.** The hub nodes in the PPI subnetwork constructed by the DEGs related to inflammatory cytokines and C-C motif chemokines

Rank	Node	Locus	Gene description	Degree
1	IL6	7p15.3	interleukin 6	61
2	CXCL12	10q11.21	C-X-C motif chemokine ligand 12	38
3	CCL2	17q12	C-C motif chemokine ligand 2	33
4	IL15	4q31.21	interleukin 15	23
5	CCL11	17q12	C-C motif chemokine ligand 11	13
6	IL33	9p24.1	interleukin 33	12
7	CCL7	17q12	C-C motif chemokine ligand 7	11
8	CD40	20q13.12	CD40 molecule	7
9	CSF1	1p13.3	colony stimulating factor 1	7
10	EGF	4q25	epidermal growth factor	7

**Table 5.** The hub nodes in the PPI subnetwork constructed by the DEGs related to the regulation of angiogenesis

Rank	Node	Locus	Gene description	Degree
1	EGF	4q25	epidermal growth factor	55
2	HGF	7q21.11	hepatocyte growth factor	42
3	FGF2	4q28.1	fibroblast growth factor 2	40
4	ERBB2	17q12	erb-b2 receptor tyrosine kinase 2	34
5	CCL2	17q12	C-C motif chemokine ligand 2	33
6	SERPINE1	7q22.1	serpin family E member 1	24
7	MMP3	11q22.2	matrix metallopeptidase 3	21
8	ANGPT2	8p23.1	angiopoietin 2	16
9	CXCL12	10q11.21	C-X-C motif chemokine ligand 12	8
10	IL6	7p15.3	interleukin 6	8

### Quantitative analysis of ESCs-secreted protein levels of IL-6 and EGF

**Figure 7.**
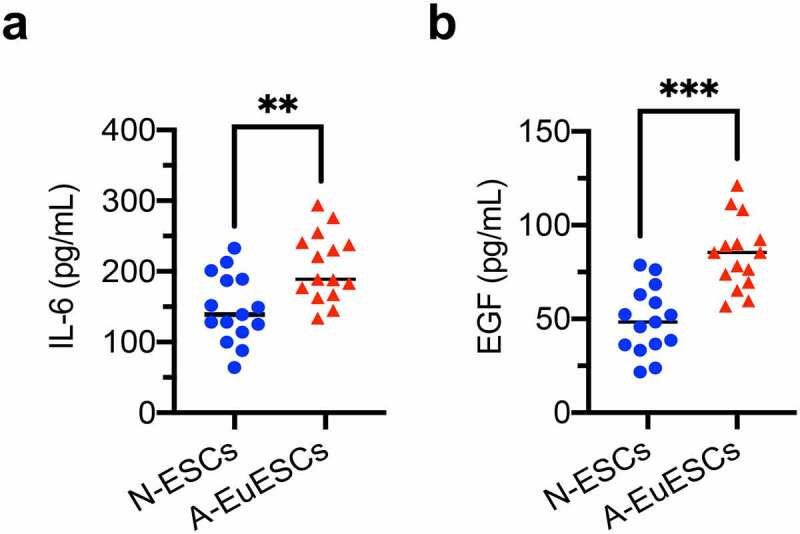
ESCs-secreted protein levels of hub genes in the cell culture supernatants ESCs-secreted protein levels in the cell culture supernatants from N-ESCs (n = 15) and A-EuESCs (n = 15) were determined using ELISA kits. (a) The concentration of IL-6 in the cell culture supernatants. (b) The concentration of EGF in the cell culture supernatants. *P < 0.05, *P < 0.01, *P < 0.001, A-EuESCs *vs*. N-ESCs.

To further detect ESCs-secreted protein levels of IL-6 and EGF in the cell culture supernatants, two ELISA kits were used. The protein levels of IL-6 and EGF in supernatants were significantly higher in A-EuESCs than those in N-ESCs (Figure 7(a,b)). These results further confirmed that IL-6 and EGF were key hub genes in the subnetworks of the DEGs related to the regulation of inflammatory cytokines/chemokines and angiogenesis.

## Discussion

To date, no study has directly investigated the transcriptomic profile of EuESCs from women with clinically significant AM. Given the cyclical changes in the uterine endometrium [41,42], the A-EuESCs and N-ESCs were collected in the mid-secretory phase of the menstrual cycle, as confirmed by histopathological examination, for RNA-Seq. Hence, our study presented the first genome-wide view of the gene expression profiles in secretory EuESCs from female patients with AM.

In the present study, we compared the transcriptomic profiles between A-EuESCs and N-ESCs. The potential functions of dysregulated genes in A-EuESCs and N-ESCs, were predicted through the GO and KEGG pathway enrichment analyses. The GO enrichment analysis revealed that the DEGs in N-EuESCs and A-EuESCs were mainly enriched in cell-cell adhesion, angiogenesis, extracellular matrix organization, chemotaxis, etc. Meanwhile, the KEGG pathway enrichment analysis revealed that the abovementioned DEGs were mainly enriched in cytokine-cytokine receptor interaction, MAPK signaling pathway, PI3K-AKT signaling pathway, and chemokine signaling pathway.

The three tandem-arrayed protocadherin (PCDH) gene clusters, including Pcdh-α, Pcdh-β, and Pcdh-γ, play fundamental roles in the development of the vertebrate central nervous system [43]. A growing body of evidence suggested that PCDHs are widely involved in the pathogenesis and progression of multiple types of cancers by enhancing invasion and metastasis [44,45]. According to the results of chord plot analysis, several PCDH-β family member genes (*PCDHB3, PCDHB4, PCDHB5, PCDHB9, PCDHB13* and *PCDHB16*), with a particularly pronounced down-regulation in A-EuESCs were directly related to calcium-dependent cell-cell adhesion. Downregulation of PCDH-β genes may affect invasion and migration of A-EuESCs by regulating intercellular adhesion and cell spreading. Meanwhile, 2 known genes, including *FOXC2* and *SERPINE1*, with a particularly pronounced up-regulation in A-EuESCs were directly related to in extracellular matrix organization. Up-regulation of these genes may promote cell invasion and migration by degrading extracellular matrix. In addition, we found that the chemotactic genes, including *IL-6, LPAR1, NTF3, CCL26, PTK2B, ADGRA2, ANGPTL4*, and *SERPINE1* were expressed through up-regulation in A-EuESCs, suggesting that A-EuESCs may have more movement tendency than N-EuESCs.

Previous studies have shown that inflammation was accumulated in the eutopic endometrium compared with in the control endometrium [46,47], which was supported by the results of the comparison between the A-EuESCs and N-ESCs in the present study. The PPI subnetworks showed that IL-6 acted as the most significant hub gene and interacted with several important inflammatory cytokines and chemokines. Recently, Xiang et al. has reported the increased mRNA expression of SERPINE1 in both eutopic and ectopic endometrium compared with that in controls during proliferative and secretory phase, while the altered expression of SERPINE1 in cellular components of endometrial tissues remained elusive [17]. Remarkably, our study further demonstrated that SERPINE1 was significantly upregulated in A-EuESCs compared with N-ESCs.

It has also been shown that angiogenesis participates in the pathophysiology of abnormal uterine bleeding and subfertility in AM [48]. Wang et al. demonstrated that A-EuESCs treated with β-estradiol presented stronger pro-angiogenetic capacities, accompanied by the increased expression levels of VEGFB and ANGPTL4 proteins [14]. Furthermore, our chord plot analysis revealed that upregulated genes of *FOXC2, TLR3, GATA6, HGF, IL-6, PTK2B, ANGPTL4*, and *HIF1A* were involved in the regulation of angiogenesis (Figure 5). Importantly, the PPI subnetworks showed that EGF acted as most significant hub gene and interacted with other angiogenic factors (HGF, ERBB2, FGF2 and SERPINE1) (Figure 6). These results demonstrated that A-EuESCs have the characteristics of pro-angiogenic activity compared with N-ESCs. Besides, the recent scRNA-seq analysis has shown that the cell motility, cell proliferation, angiogenesis, and inflammation terms were enriched in eutopic endometrium versus normal endometrium. The DEGs were mainly functioned in angiogenesis and cell mobility-related cytoskeleton regulation and chemotaxis. However, there is no information related to stromal cell subpopulation [18]. Hence, the overlapping of our findings with the previous studies may reflect the universal mechanisms underlying the AM and the reliability of our data.

In summary, these findings, for the first time, revealed the overall characteristics of A-EuESCs from the perspective of transcriptomic profiles. Besides, there are several limitations in our study. First, endometrial samples analyzed herein were from the mid-secretory phase of menstrual cycle, and study of gene dysregulation during proliferative phase may increase the understanding of abnormalities of A-EuESCs in the pathogenesis of AM. Second, the sample size was small, and further study should be conducted with a larger sample size. Third, the dysregulated genes were only validated through qRT-PCR and ELISA in ESCs, and no validation was carried out by in situ biospecimens. Last but not least, the biological functions of several DEGs, including *HOXC8, TCF21, CACNG7, FOXC2, SERPINE1, ADGRA2, NTF3, TLR3, GATA6, KCTD8* and *DTX1* in the pathophysiology of AM remained unclear and should be further explored by *in vitro* and *in vivo* studies.

## Conclusions

The present study provided an important basis to prior focal studies that revealed fundamental abnormalities in A-EuESCs in women with AM. The findings of the DEGs lay a foundation for further investigation to elucidate the mechanisms of AM, and the altered pathways in A-EuESCs may predispose to pro-invasion/migration and angiogenesis, which may be involved in the development of AM. The DEGs and pathways may assist scholars in the development of further efficacious therapies for AM. However, specific roles and mechanisms of the DEGs in A-EuESCs should be investigated and confirmed in the future study.

## Supplementary Material

Supplemental Material
