# Supplementary material for: Transcriptome analysis of eutopic endometrial stromal cells in women with adenomyosis by RNA-sequencing
Source: Bioengineered. 2022 May 21;13(5):12637–49. doi: 10.1080/21655979.2022.2077614 (PMC9275863; doi:10.1080/21655979.2022.2077614)
Supplement: Supplemental Material [file KBIE_A_2077614_SM2565.zip › Supplementary Table 1.docx]

**Supplementary Table 1** The sequences of primers used for qRT-PCR analysis

| **Gene symbol** | **Direction** | **Sequence (5’→3’)** |
| --- | --- | --- |
| HOXC8 | Forward | CCAAATACAAAGCCGGCGAG |
|  | Reverse | GCGCCTCGTAGCCATAGAAT |
| IL33 | Forward | GCCTTGTGTTTCAAGCTGGG |
|  | Reverse | CCAAAGGCAAAGCACTCCAC |
| MYH1 | Forward | AGCTGAGGTGTAACGGTGTG |
|  | Reverse | TCTAGGAGCCCCAGAAGACC |
| TCF21 | Forward | CCAACTGCGAGAATGGGTCT |
|  | Reverse | AGCTTGGAGAGCTTGGTGTC |
| ADAMTSL1 | Forward | CATCGGCTGAGTGCTACGAT |
|  | Reverse | GCTTGTATCCGTCACTGGCT |
| MYH2 | Forward | TTCTCTGGGGCTCAAACTGC |
|  | Reverse | TGGGTACTCCTGAGGTTGGT |
| CCL7 | Forward | ATCCCTAAGCAGAGGCTGGA |
|  | Reverse | GTCCTGGACCCACTTCTGTG |
| CCL11 | Forward | AGTGGCAAATGTCCCCAGAA |
|  | Reverse | ACTTCATGGAATCCTGCACCC |
| ACAN | Forward | TTCCGCTGGTCAGATGGACA |
|  | Reverse | CGTTTGTAGGTGGTGGCTGTG |
| CACNG7 | Forward | CTACGGGTGGTCTTTTGCCT |
|  | Reverse | ATTTGGATGGACACGTCGCT |
| SLC6A2 | Forward | CAGGCACCTCCATTCTGTTT |
|  | Reverse | GCGGCTTGAAGTTGATGATGCTG |
| MYCN | Forward | ATGACTTCTACTTCGGCGGC |
|  | Reverse | CCACAGCTCGTTCTCAAGCA |
| KCTD8 | Forward | GAAGTACACGTCCCGCTTCT |
|  | Reverse | TTTCTGAGGTGGTCGGAAGA |
| NPBWR1 | Forward | GTACCAGTTGTCTACGCGGT |
|  | Reverse | GAACAGGTTGGTGACGGTCT |
| NKD2 | Forward | TTGCAGGATGGAGGGTGAAC |
|  | Reverse | CTTGCACAGGAGGGGACC |
| F13A1 | Forward | TCAGAAACTTCCAGGACCGC |
|  | Reverse | AGGTGAACGCTCGTGACATT |
| MMP7 | Forward | TACCCATTTGATGGGCCAGG |
|  | Reverse | AGACTGCTACCATCCGTCCA |
| LPL | Forward | CCGCCGACCAAAGAAGAGAT |
|  | Reverse | TAGCCACGGACTCTGCTACT |
| COCH | Forward | TCCAATACAGGAAAAGCCTTGA |
|  | Reverse | ATGCCTGCTTCCTCGATGTC |
| DTX1 | Forward | GCTGATGCCTGTGAATGGTC |
|  | Reverse | GCAGGTCGATGATGTAGGGC |

| **Gene symbol** | **Direction** | **Sequence (5’→3’)** |
| --- | --- | --- |
| ADAM12 | Forward | CTCGCTCGAAATTACACGGT |
|  | Reverse | CAGCGAGGTTTGGTGTGTTG |
| SERPINE1 | Forward | CACTCTTGTACTGCCTGCCA |
|  | Reverse | TGCACACTGTTTCTGGGGAG |
| ADGRA2 | Forward | TGACCTCTGCTGTCTTTGCG |
|  | Reverse | CGATCAAATAGAACCGGAGCA |
| ANGPTL4 | Forward | TCTCTGGAGGCTGGTGGTTT |
|  | Reverse | GATCAACATGGTGGTGGCCT |
| IL6 | Forward | AGTGAGGAACAAGCCAGAGC |
|  | Reverse | AGCTGCGCAGAATGAGATGA |
| NTF3 | Forward | TGGTTACTTTTGCCACGATCT |
|  | Reverse | ATTGAGCGAGTCTTCTGGCA |
| CCL2 | Forward | GATGCAATCAATGCCCCAGTC |
|  | Reverse | TTTGGGACACTTGCTGCTGG |
| TLR3 | Forward | CCTTTTGCCCTTTGGGATGC |
|  | Reverse | TGAAGTTGGCGGCTGGTAAT |
| GATA6 | Forward | CTCAGTTCCTACGCTTCGCA |
|  | Reverse | GCTTGGTCGAGGTCAGTGAA |
| HGF | Forward | ACCCTGGTGTTTCACAAGCA |
|  | Reverse | GCAAGAATTTGTGCCGGTGT |
| KDR | Forward | CAAGTGGCTAAGGGCATGGA |
|  | Reverse | ATTTCAAAGGGAGGCGAGCA |
| GAPDH | Forward | GAGCCACATCGCTCAGACAC |
|  | Reverse | CATGTAGTTGAGGTCAATGAAGG |

**-Continue-**
