# Supplementary figures and images for: Transcriptome analysis of eutopic endometrial stromal cells in women with adenomyosis by RNA-sequencing
Source: Bioengineered. 2022 May 21;13(5):12637–49. doi: 10.1080/21655979.2022.2077614 (PMC9275863; doi:10.1080/21655979.2022.2077614)

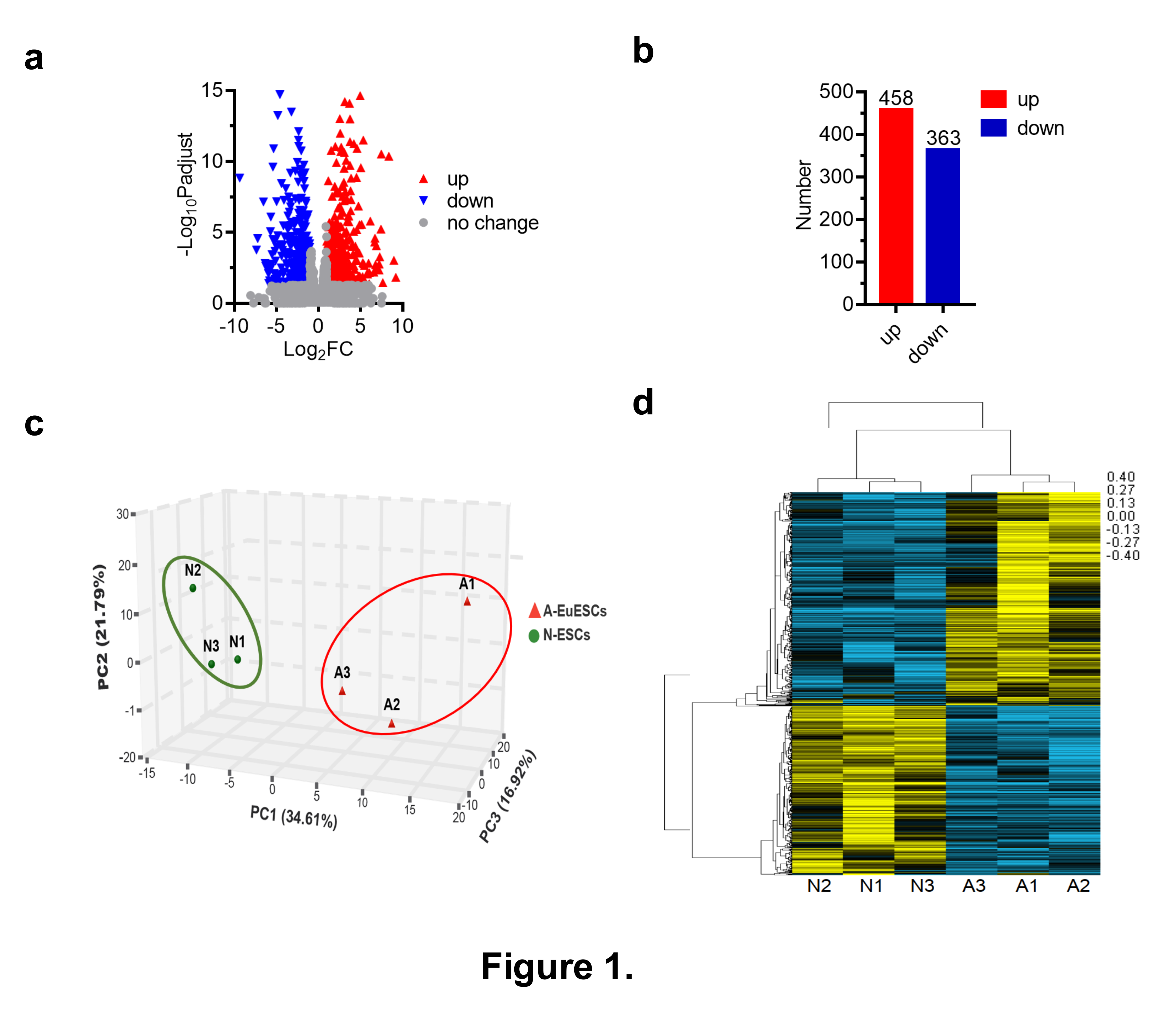

Supplement: Supplemental Material [file KBIE_A_2077614_SM2565.zip › Supplementary Figure 1 (1).tif]
